# Nutrition and Muscle Strength, As the Key Component of Sarcopenia: An Overview of Current Evidence

**DOI:** 10.3390/nu11122942

**Published:** 2019-12-03

**Authors:** Sian Robinson, Antoneta Granic, Avan Aihie Sayer

**Affiliations:** 1 AGE Research Group, Translational and Clinical Research Institute, Faculty of Medical Sciences, Newcastle University, Newcastle upon Tyne NE4 5PL, UK; antoneta.granic@newcastle.ac.uk (A.G.); avan.sayer@newcastle.ac.uk (A.A.S.); 2 NIHR Newcastle Biomedical Research Centre, Newcastle upon Tyne Hospitals NHS Foundation Trust and Newcastle University, Newcastle upon Tyne NE4 5PL, UK; 3 Newcastle upon Tyne Hospitals NHS Foundation Trust, Newcastle upon Tyne NE7 7DN, UK

**Keywords:** diet, muscle strength, sarcopenia, aetiology, ageing, review

## Abstract

Much has been achieved by recent research to increase understanding of the links between nutrition and muscle health. Focusing on muscle strength as the key component of sarcopenia, the aim of this overview was to evaluate its links to nutrition, both to variation in habitual diets in older populations, as well as considering supplementation effects in trials. A main message from the reviewed studies is that while many provide suggestive evidence of benefits of higher nutrient intakes and diets of higher quality, findings are inconsistent, and data on muscle strength are often lacking. To assess the potential of optimising diets as a strategy to promote and maintain muscle strength, gaps in current evidence need to be addressed. These include the need for (i) better understanding of individual differences in responsiveness to dietary change, and the need for targeted nutritional support; (ii) clearer distinction between protective and therapeutic actions of diet; and (iii) definition of the role of dietary patterns and their influence on muscle strength, to allow effects of changes in food consumption to be evaluated—particularly when combined with physical activity. Development of this evidence is needed to enable translation into appropriate dietary recommendations for older populations.

## 1. Sarcopenia

Sarcopenia is a skeletal muscle disorder that is characterised by the loss of strength and mass together with impairment in physical function. Although more common among older people, and seen principally as an age-related condition, it is also found in younger adults and, importantly, is influenced by risk factors operating across the lifecourse [1]. Sarcopenia is associated with an array of poor health outcomes that include frailty, hospitalisation and mortality [1,2,3]. It was formally recognised with an International Classification of Diseases-10 code in 2016.

In the decades since its first description [4], sarcopenia has become a focus of intense research activity—both to understand its underpinning pathophysiology as well to consider translation of this knowledge into prevention and treatment strategies [1]. The importance of exercise and physical activity [5,6] as well as nutrition [7,8], as modifiable influences on muscle health and function, has long been known. There is a large body of evidence that shows clear benefits of exercise interventions both to prevent and treat losses of muscle strength and mass in older people [9,10]; in particular, the efficacy of resistance exercise training is well established [11]. However, in comparison, the links with nutrition, and the potential of dietary change to improve muscle outcomes, are not well understood [1]. Although much has been achieved in research in the past decade, and the evidence base has developed substantially, the nature of optimal diets to support the maintenance of muscle strength and mass in older age has yet to be defined. Furthermore, outside the context of malnutrition, which has recognised overlap with sarcopenia [12], the impact of poor habitual diets and marginal nutrient intakes, as described in older populations, is not certain [13]. Given the prevalence and the impact of sarcopenia, there is a pressing need for this evidence to enable translation into appropriate dietary recommendations for older populations to optimise muscle health.

**Figure 1:**
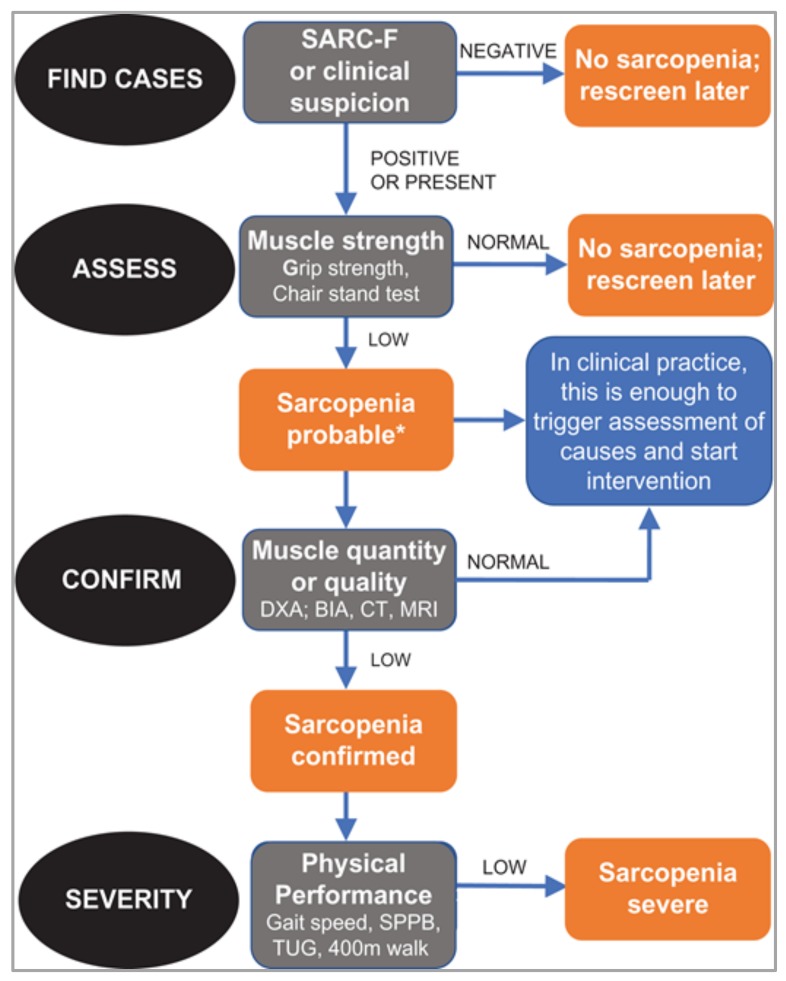
Sarcopenia: European Working Group on Sarcopenia in Older People (EWGSOP2) algorithm for case finding, making a diagnosis and quantifying severity in practice (*considering other reasons for low muscle strength such as depression, stroke, balance disorders, peripheral vascular disorders) (DXA: dual-energy X-ray absorptiometry, BIA: bioelectrical impedance analysis, CT: computed tomography, MRI: magnetic resonance imaging, SPPB: short physical performance battery, TUG: timed-up-and-go test) [14]. Permission has been obtained from Oxford University Press.

One of the challenges in understanding current evidence of the role of nutrition is the number of different aspects of muscle health that have been considered as outcomes, both in observational studies as well as in intervention trials. These include muscle mass and muscle strength (measured in different ways), measures of physical performance, as well as sarcopenia that, in turn, may have been diagnosed according to different criteria. To researchers outside the field, there is a risk that these different muscle parameters may be seen to be equivalent, and even to be considered interchangeably. So, an important development has been the recent publication of revised guidelines on the definition and diagnosis of sarcopenia by the European Working Group on Sarcopenia in Older People [14]. The new guidelines, which aim to improve consistency in the identification of sarcopenia in clinical care, identify muscle strength as the key characteristic of sarcopenia with low muscle strength leading to a diagnosis of probable sarcopenia; subsequent identification of low muscle mass in individuals who have low muscle strength is used to confirm its presence and, when physical performance is also poor, its severity (Figure 1).

As well as a tool to improve identification of sarcopenia, this new guidance may also offer a useful structure within which to evaluate influences on muscle health, including the effects of differences in diet. The aim of this review was therefore to consider current evidence on the role of nutrition as an influence on sarcopenia but, to align with the EWGSOP2 guidance [14], to focus primarily on its links to muscle strength. We therefore have not included detail on other muscle outcomes, although these were reported in some of the reviewed studies.

## 2. Nutrition in Older Age

Alongside lower levels of physical activity and a reduction in energy needs in older age, food consumption declines and energy intakes fall. The effect of age on energy intake is significant. For example, in a recent meta-analysis of studies of healthy adults, this amounted to a difference of approximately 20% when comparing younger (aged 26 years) and older (aged 70 years) groups [15], and steeper declines in intake have been reported among even older adults [16]. The drivers of low food intake include the effects of age-related changes in sensory perception [17], poor oral health [18] and impaired appetite [19], that can act to reduce meal size and eating frequency and may also affect food choice. In particular, appetite loss, described as the ‘anorexia of ageing’, is often reported and is a key determinant of nutritional risk [20]. However, influences on diet in older age are complex and, in addition to these physiological changes, there is an array of wider contextual and personal factors that also impact on dietary intake [21]. Notably, in parallel with declining energy intake, intakes of other nutrients, including protein and micronutrients, are also likely to fall [22]. As requirements for some nutrients do not change, or may even increase in older age, without greater consumption of nutrient-dense foods it can become more challenging for older adults to meet nutrient needs [23]. This underlines the need for a diet of adequate quality—potentially at the same time as food access and preparation are becoming more challenging and diets more monotonous, contributing to nutritional risk. Although the prevalence of poor diets varies across studies of older populations, consistent with this concern, low diet quality is common in developed settings [24] and malnutrition rates are high [25].

## 3. Mechanistic Considerations

There is now a body of evidence that links poor nutrition to adverse effects on muscle in older age, suggesting that strategies that support older adults to maintain nutrient intake could help to preserve muscle strength and mass—and could be effective both to prevent and treat sarcopenia. Although the underpinning physiology is not fully understood, a number of possible mechanisms are recognised. Firstly, low food consumption can lead to insufficient energy intakes; the ensuing loss of body weight is due not only to depletion of stored fat but also to the catabolism of muscle, leading to reduced muscle mass [26]. Secondly, protein requirements may be increased in older age. Apart from providing amino acids, the consumption of dietary protein is an anabolic stimulus that has direct effects on muscle protein synthesis. As this response has been shown to be blunted among older adults, their protein intakes may need to be relatively greater to maintain nitrogen balance and to prevent losses of muscle mass and strength [7,27]. Thirdly, there may be important anti-oxidant effects of some dietary components. Oxidative stress, arising from the accumulation of reactive oxygen species (ROS) can damage biomolecules in muscle [28] and, via effects on signalling pathways, is also linked to inflammation [29]. Whilst normally counterbalanced by effects of endogenous antioxidants, the role of exogenous antioxidants may be significant [30]; ensuring sufficient intakes of dietary antioxidants in older age, such as carotenoids and selenium, may therefore offer protection against oxidative damage to muscle tissue. Fourthly, the links between inflammation and sarcopenia point both to the potential benefits of dietary factors that have anti-inflammatory effects, such as *n*-3 fatty acids, as well as the importance of dietary influences on adiposity, particularly visceral adiposity, that contribute to a low-grade inflammatory state [13]. Supplementation with *n*-3 long chain polyunsaturated fatty acids (LCPUFAs) has a lowering effect on CRP, IL-6 and TNF-α levels; manipulation of the balance and dietary content of *n*-3 and *n*-6 LCPUFAs may therefore have potential as an approach to prevent or treat sarcopenia [31]. Finally, there is suggestive evidence of influences of other dietary components, but for which understanding of the underpinning mechanisms is currently less clear. The best example of these may be vitamin D, for which there is considerable interest in the links between status and muscle function [7]. But recently, with increased understanding of the role and influence of the gut microbiota on health, effects on skeletal muscle function, potentially via effects on anabolic resistance, have also been suggested [32]. These effects could be important and, as diet is a key influence on microbial composition in the gut [33], merit further study.

## 4. Nutrition and Muscle Strength

The growth in evidence that examines links between diet and muscle outcomes in older populations has enabled collation of findings to establish consistency across studies and, in some cases, meta-analyses to evaluate effect size. The following sections provide an overview of current evidence on muscle strength, focusing particularly, where available, on recent systematic reviews and meta-analyses.

### 4.1. Observational Evidence

#### 4.1.1. Protein

There has been intense interest in the role of protein in the pathogenesis of sarcopenia, linked to anabolic resistance, and the possibility that dietary requirements for protein are increased in older age. There is some observational evidence that has examined links between habitual protein intakes and muscle strength. Most recently, a systematic review and meta-analysis of observational evidence was published by Coelho-Junior and colleagues [34]. The authors examined differences between study participants, aged 60 years or older, who had high (≥1.0 g/kg body weight) or very high (≥1.2 g/kg) daily protein intakes with those with lower levels of intake (<0.8 g/kg; 0.8–0.99 g/kg). Upper limb muscle strength was assessed by isometric handgrip strength; lower limb strength by measured chair rise time or knee extensor strength. In total, six studies reporting strength outcomes were evaluated in meta-analyses. Overall, although some differences were described among older adults who had high protein intakes, these were non-significant. Notably, the lack of differences in muscle strength differed from findings for physical performance in this review; for example, faster walking speed was found among older adults whose protein intakes were higher [34].

However, other observational data on muscle strength, published around the time of this review, yielded different findings; positive effects of higher protein intakes were found in cross-sectional studies of younger and older adults. The largest of these studies was the UK Biobank cohort of 146,816 adults aged 40–69 years [35]. In this analysis, graded increases in grip strength (expressed as kg/kg body weight) across the range of daily protein intakes from <0.8 g/kg to 2 g/kg were observed. Consistent with this finding, a positive association between protein intake and muscle strength (expressed as kg/BMI) was also reported in a recent study of a diverse cohort (2468 adults aged 33 to 71 years) in the US [36]. In both studies, the associations were seen in men and women, and adjusted for a wide range of covariates, including age, ethnicity, social status, smoking and comorbidities. The apparent inconsistency when comparing these findings with the meta-analyses is unlikely to be due to differences in the ages of participants, as a lack of independent associations between total protein intake and muscle strength has been reported in other cross-sectional analyses of data from cohorts with comparable age ranges [37,38,39], although in these studies, uncorrected measures of strength (kg) rather than corrected (per kg or BMI) were reported. Such observational findings may simply differ across cohorts, depending on differences in participant characteristics, such as their habitual diets, nutritional status or levels of physical activity. For example, in a recent cross-sectional analysis of data from an older cohort in the UK (the Newcastle 85+ Study, 722 community-dwelling men and women), consistent with the effects described above [35,36], a low protein intake (<1 g/kg) was found to associated with lower grip strength (uncorrected, kg) [40]. Inconsistencies in longitudinal effects are also apparent; for example in the Newcastle 85+ study, no association was found between protein intake and subsequent decline in strength [40], whereas protective effects of higher levels of protein intake have been reported in other longitudinal cohorts [37].

#### 4.1.2. Antioxidant Nutrients

In comparison with studies of dietary protein, there have been far fewer investigations into the role of dietary antioxidants, despite known links between markers of oxidative damage, such as higher serum concentration of protein carbonyls and lower muscle strength [41]. There are some epidemiological studies that support the possibility that variation in dietary antioxidant intake may influence muscle strength in older age [42]. The best known evidence comes from the InCHIANTI (Invecchiare in Chianti) and WHAS (Women’s Health and Aging Study) cohorts, in which lower blood concentrations of selenium, alpha-tocopherol and carotenoids have been shown to be associated with lower muscle strength in cross-sectional analyses [43,44,45] and, in InCHIANTI, lower carotenoid status predicted greater loss of strength (handgrip, knee and hip) over a follow-up period of six years [46]. However, relatively little progress has been made in understanding the role of habitual dietary antioxidant intakes and their influence on muscle strength in the decade since. For example, in a large study of community-dwelling adults (aged 40–80 years) self-reported use of vitamin C and E supplements was not related to grip strength or to change in strength over a 5-year follow-up period [47]. Also, in a recent systematic review that considered the influence of dietary selenium on sarcopenia, only five studies were included; whilst two showed differences in intake between sarcopenic and non-sarcopenic older adults, no evidence of effects of selenium on muscle strength was reported [48]. Given the recognised role of oxidative damage and its effects on muscle cells [30], this may be surprising. However, as much has been learnt in this time about the potential benefits of patterns of diet that provide higher habitual intakes of many of these nutrients (Section 4.1.6), more needs to be known about the potential influence of dietary antioxidants.

#### 4.1.3. Anti-Inflammatory Nutrients

Chronic low-grade inflammation is implicated in the aetiology of a range of age-related conditions, including sarcopenia. For example, among older participants in the Hertfordshire Ageing Study, a greater ‘inflammaging’ burden, defined by blood inflammatory biomarker concentrations, was shown to predict lower muscle strength at 10-year follow-up [49]. Recent research has focused on the anti-inflammatory effects of diet—particularly on the role of LCPUFAs [31]. At present, there is little observational evidence that links differences in habitual LCPUFA intake to muscle strength, although in a cross-sectional analysis, greater oily fish consumption (rich in *n*-3 LCPUFAs) was found to be associated with greater grip strength among older community-dwelling adults—importantly, a difference that was not observed in relation to consumption of white fish [50]. Other studies have also reported a link where higher reported intakes of *n*-3 fatty acids [51] and higher status (total plasma phospholipid *n*-3 polyunsaturated fatty acids) were related to measures of muscle strength [52], although in both of these studies the observed differences were mainly attenuated following adjustment for confounders. In a recent review of the effects of *n*-3 fatty acids, Rossato and colleagues comment on the inconsistency of the available observational evidence, suggesting that the lack of independent associations in the adjusted statistical models that link *n*-3 intakes and status with muscle strength may be evidence of their indirect rather than direct effects [53].

One challenge in understanding the role of LCPUFAs from observational evidence is the collinearity of dietary constituents—such that older adults who have higher intakes may differ in their habitual diets, with LCPUFAs acting as markers for other dietary constituents, and it is these that are causally related to muscle health [7]. For example, high LCPUFA intakes may just indicate ‘healthier’ diets that have a higher content of bioactive components with anti-inflammatory actions, such as dietary flavonoids [54,55]. The development of a dietary inflammatory index (DII) is therefore important, as this enables the overall inflammatory potential of the diet to be evaluated by summing the inflammatory effects of a number of individual dietary components, that include β-carotene, fatty acids, vitamins A, C and E and zinc [56]. Cervo and colleagues have recently described prospective associations between DII, assessed in this way, and measures of musculoskeletal health for the first time, using data from 1098 participants in the Tasmanian Older Adult Cohort Study. However, while the authors found negative effects on bone health associated with a more proinflammatory diet, apart from an unexpected increase in lower limb muscle quality, there were no associations with muscle strength [56].

#### 4.1.4. Vitamin D

The mechanisms that link vitamin D insufficiency to sarcopenic changes in older age are not fully understood. However, as proximal muscle weakness is a feature of clinical vitamin D deficiency, and low vitamin D status is common in many older populations, there is considerable interest in the potential benefits of therapeutic interventions to improve status. The interest in vitamin D has led to a sizeable body of research to develop understanding of its links to muscle health [50]. Much of this research is interventional (summarised in Section 4.2.4); less is known about the differences observed in vitamin D status across older populations and their implications for muscle strength. Overall, the messages from the observational evidence of vitamin D status and muscle strength appear to be mixed [57]. For example, low vitamin D status at baseline was associated with a greater loss of grip strength over a three-year follow-up period among older participants in the Longitudinal Aging Study Amsterdam [58]; in contrast, among older participants in the Health ABC Study, whilst baseline status was associated with lower muscle strength, it did not predict losses of muscle strength over the following four years [59]. Such inconsistencies between studies, which may arise from differences in study design and population characteristics, also persist in more recent research, in which both positive associations [60], as well as lack of associations [61,62], with vitamin D have been reported.

#### 4.1.5. Foods

Apart from the recognised limitations of observational data to draw causal inferences, the collinearity between dietary components challenges interpretation of studies of single nutrients. There are therefore advantages to using a whole-foods approach. In particular, this allows consideration of grouped dietary constituents that are found together in the same foods, and, while potentially of less value to understand underpinning mechanisms, it has important implications for public health considerations. However, to date there have been few studies of whole foods and there is currently little observational evidence of effects on muscle strength. There is some research to suggest benefits of higher consumption of fruit and vegetables [50,63] and dairy foods [64]. For example, in an Australian study of 1456 older women (aged 70–85 years) higher dairy consumption (milk, yogurt and cheese) was associated with greater grip strength. However, evidence is very limited; further data from new studies of whole foods are needed to be able assess their significance for the protection and promotion of muscle health in older age.

#### 4.1.6. Dietary Patterns

Greater progress has been made over the past decade in the use of dietary patterns to understand the role of habitual diets in the aetiology of sarcopenia [65]. In common with a whole-foods approach, patterns analysis enables evaluation of the overall effects of qualitative differences in diet, taking account of potential synergistic and other interactions between dietary constituents. There is now a sufficient number of studies that have described dietary patterns in relation to the components of sarcopenia to enable collation of this evidence. The first systematic review of the effects of differences in diet ‘quality’ (describing a ‘healthier’ profile of foods) was reported by Bloom and colleagues in 2018 [66]. The review included 23 studies of older adults; 11 reported data on muscle strength. As diet quality was assessed using a priori scores (*n* = 8) and patterns analysis (*n* = 3), the differences in its definition prevented the authors from including the studies in a meta-analysis. Of the 11 studies reviewed, only five found positive associations with muscle strength, and patterns of association were not consistent across types of study design (cross-sectional vs. longitudinal) or between genders. This lack of consistency was in contrast to clearer evidence of links between higher diet quality and better measured physical performance [66]. The lack of common patterns of association between diet quality and different muscle outcomes echoes findings of recent reviews of the effects of the Mediterranean dietary pattern, with some mixed effects shown for strength, frailty and functional disability among older population groups [67,68], although in a new study of older women (aged 60–85 years), higher muscle (grip) strength was found among participants who complied with a Mediterranean dietary pattern [69]. Further evidence to improve understanding of the importance and role of dietary patterns for muscle health is needed. As habitual diets of higher quality should provide greater intakes of a range of nutrients and non-nutrients that are linked to muscle health, and may also be linked to beneficial effects on gut microbial composition [32,33], their potential protective benefits in older age could be significant [7].

### 4.2. Dietary Interventions

While existing observational evidence of links between nutrition and muscle strength in older age may not yield clear messages, the prevalence of poor nutrition in older populations, together with established mechanistic links between nutrition and muscle function, suggest that intervention to increase nutrient intake has potential to improve outcomes—at least among older individuals who are at nutritional risk. There is now a sizeable body of literature on the effects of nutrient supplementation, although there are challenges in collating the findings of these studies arising from differences in study design, the nature of populations studied, nutrients provided (singly or in combinations), the dose and duration of supplementation, and most notably, whether combined with exercise training or not. To date there have been few studies of whole food provision as a device to increase nutrient intake, and even less is currently known about the efficacy of making overall changes in dietary patterns to achieve gains in muscle strength.

#### 4.2.1. Protein and Amino Acid Supplementation

The most studied types of dietary supplement to improve muscle outcomes are proteins and amino acids. In the systematic review for the first report of the European Working Group on Sarcopenia in Older People, 11 of the 12 included studies contained protein, essential amino acids or HMB (a leucine metabolite) [6]. The findings from this review, consistent with the observational evidence of differences in habitual protein intake (Section 4.1.1), showed the effects of supplementation to be mixed. More recent systematic reviews of protein/amino acid supplementation studies of older adults have yielded similar findings [70,71,72]. For example, in the first of these reviews, although some evidence of benefit was found in individual studies of older adults, pooled analyses showed that the overall effects on muscle strength were not significant [70]; in the second, meta-analyses of data from supplemented non-frail community-dwelling older adults showed that while there was a tendency for grip strength to increase in protein-supplemented participants (standardised mean difference: 0.58; 95% CI: −0.08 to 1.24, *p* = 0.08), there were no differences in lower extremity strength [71].

However, the most recent systematic review and meta-analysis of data from 32 large RCTs of nutritional supplementation of older adults, reported by Veronese and colleagues (evidence published up to September 2018) [72], showed that supplementation with protein or amino acids improved handgrip strength (*n* = 7 studies; SMD 0.24, 95% CI 0.07 to 0.41). Positive effects were also seen in trials of ‘multinutrient’ supplements, the majority of which also included protein or amino acids (*n* = 6 studies; SMD 0.41, 95% CI 0.06 to 0.76). In contrast, few associations were found with measured physical performance. An important feature of this review was the separate consideration given to the effects of supplementation among trial participants who differed in health status. For chair rise time and handgrip strength, there were significant interactions, such that the improvements in muscle strength were seen particularly among participants affected by frailty or sarcopenia [72]. This finding may be key to understanding inconsistencies in current evidence but is generally underexplored. More needs to be known about individual responsiveness to dietary supplementation, and its determinants, to inform future nutritional strategies to promote muscle health in older age.

#### 4.2.2. Antioxidant Supplementation

The observational evidence (Section 4.1.2), together with the recognised role of oxidative stress in the aetiology of sarcopenia, point to the potential of antioxidant supplementation to promote muscle health in older age [73]. However, there have been few trials of supplementation in older populations, and such benefits remain uncertain [74]. There has been some experimental and clinical research that has focused on supplementation effects on muscle responses to exercise but, to date, findings have been inconsistent [30]. This may partly reflect the dual functions of ROS, such that low levels are linked to increased muscle force, with high levels linked to declines in physical performance [73]—limiting the success of interventions that aim to suppress their activities. Furthermore, a recent trial of high-dose vitamin C and E supplementation in older men has raised concerns as it showed that some of the adaptations to strength training were blunted following supplementation [75]. At present, there is insufficient evidence to consider the use of antioxidant supplements as a nutritional strategy to prevent age-related losses of muscle strength and mass, or to treat sarcopenia [73].

#### 4.2.3. Anti-Inflammatory Nutrient Supplementation

Supplementation with *n*-3 LCPUFAs can affect protein synthesis via mTOR signalling as well as impact on inflammatory responses [7], and their use, to prevent and treat sarcopenia, which is a focus of current research. In a recent review, Dupont and colleagues summarised the findings of four studies of supplementation of older adults; four further studies combined supplementation with physical exercise (reviewed in Section 4.3) [31]. Overall, the effects of supplementation appeared promising, with measured differences found in muscle protein synthesis and physical performance. However, the effects of *n*-3 LCPUFA supplementation on muscle strength were less clear; of the trials that reported muscle strength outcomes, increases were reported in one [76], whereas no effects were found in two others [77,78]. With limited evidence to date, some inconsistencies are likely to arise from differences in dose and duration of supplementation, as well as differences in participant characteristics, and further research is needed [31].

#### 4.2.4. Vitamin D Supplementation

In comparison with antioxidant and anti-inflammatory nutrients, there is a much larger body of research that has examined the effects of vitamin D supplementation on muscle outcomes—and a number of systematic reviews and meta-analyses of this evidence have been conducted [7]. Early reviews suggested that vitamin D supplementation of older adults improved muscle strength, although effects were modest [79,80]. However, two recent reviews have not confirmed benefits of supplementation on muscle strength. In the systematic review by Rosendahl-Riise and colleagues, seven studies (published up to April 2016) were included; in the majority of these, there was no improvement in in handgrip strength, and the overall change in the meta-analysis was non-significant [81]. Consistent with this finding, the more recent meta-analysis by Veronese and colleagues also showed no overall effects on muscle strength in reviewed trials of vitamin D supplementation [72]. There may be differences in responses across studies according to the vitamin D status of the participants. For example, in a review of 17 RCTs, Stockton and colleagues found no effects of vitamin D supplementation on muscle strength among adults who had serum 25(OH)D concentrations >25 nmol/L at baseline, whereas a significant effect of supplementation on hip muscle strength was observed among deficient participants (25(OH)D < 25 nmol/L) [82]. However, in the meta-analysis reported by Rosendahl-Riise and colleagues, the observed heterogeneity in supplementation effects on strength was eliminated after exclusion of three studies of deficient participants; furthermore, their exclusion revealed a small overall gain in handgrip strength among participants who had sufficient status at baseline [81]. At present, the benefits of vitamin D supplementation to achieve improvements in muscle strength are therefore unclear.

#### 4.2.5. Changing Patterns of Food Intake

Relatively few studies have used supplementation with whole foods or manipulation of dietary patterns to improve muscle outcomes [65]. There have been some studies of milk and dairy products, although mostly these used milk/whey proteins. In a recent systematic review and meta-analysis of dairy protein effects on muscle strength, Hanach and colleagues included 14 studies; only four investigated the effects of supplementation with a whole dairy food (ricotta cheese) or milk-based protein [83]. The meta-analysis showed no overall effects on muscle strength.

Other foods of interest are fruit and vegetables. Although the effects of increasing intakes of some of their constituents (e.g., antioxidant nutrients (Section 4.2.2), dietary nitrates [84]) have been studied, there are few data on the effects of increased consumption of the whole foods. Evidence from the first RCT was reported by Neville and colleagues in 2013 [85]: older participants who had low fruit and vegetable consumption (two or fewer portions per day) were randomised either to maintain their habitual diet or, provided with free fruit and vegetables, to increase daily consumption to five or more portions, for a period of 16 weeks. Whilst there were no effects on physical performance (SPPB), there was a trend towards a gain in grip strength at the end of this period (mean change in the intervention group (2.04 ± 5.16 kg) compared with the control group (0.11 ± 3.26 kg)). Although of borderline statistical significance (*p* = 0.06), the effect size is large and suggests that there may be important benefits of increasing intake of fruit and vegetable among low consumers. The authors highlighted the need for further randomised controlled trials in older populations to examine the effects of increasing fruit and vegetable consumption for muscle health of older adults, stressing the need to ensure future trials are of adequate size and duration [85].

Greater fruit and vegetable consumption are hallmarks of ‘healthier’ dietary patterns, and the effects on muscle strength described in the RCT may be consistent with greater muscle strength seen among adults whose dietary patterns comply with this profile [69]. At present, there is almost no evidence from trials of manipulation of dietary patterns to be able to assess this, although the clear potential for improvements in muscle health in older populations deserves further exploration [65].

### 4.3. Nutrition and Exercise Interventions

The interactions between nutrition and physical activity may be important [86], and many intervention studies have combined nutritional supplementation with exercise training to enable the potential for additive or synergistic effects on muscle health to be explored. A number of reviews have collated this evidence; all point to the degree of heterogeneity across studies, particularly in terms of study design and participant characteristics, and, overall, the lack of conclusive messages that can be drawn [87,88,89].

However, some studies have suggested synergistic effects, such that exercise training may only be effective among supplemented participants, or that the training effects are enhanced in supplemented individuals. Two current examples of interest are supplementation with protein/amino acids or with *n*-3 LCPUFAs. In the most recent systematic review and meta-analysis, Hou and colleagues included 21 RCTs of older participants (published January 2004 to May 2018). Protein supplementation combined with resistance training resulted in greater gains in muscle strength, although differences in physical performance were not found [90]. Comparable effects have been described in primary studies of *n*-3 LCPUFA supplementation in which greater strength gains have been observed among supplemented older women who received training [91,92], but not in men [92]. Since the number of studies of LCPUFAs is still small, further research is needed to confirm such effects.

The combined effects of food supplementation with exercise training is very limited, although in a trial of combined exercise training with/without increased meat consumption in a group of older women, Daly and colleagues reported greater gains in muscle strength in response to resistance exercise training in the meat group, in comparison with control resistance-trained participants [93]. However, in contrast, increased consumption of fortified milk in combination with exercise training has not been shown to enhance training effects [94]. Perhaps the most encouraging data come from a recent Swedish RCT in older women, a rare example of a combined resistance exercise training and dietary intervention to improve muscle outcomes, in which the change was an alteration in dietary pattern, specifically, to increase compliance with a ‘healthier’ dietary pattern over a period of 24 weeks (based on wholegrain foods, fruits and vegetables, fish, and polyunsaturated fats from vegetable oils and nuts) [95,96]. Improvements in muscle power were seen both in the exercise and the exercise–healthy diet groups, but the gains were greater in the group that combined exercise training with a healthier pattern of diet [95]. Furthermore, combined resistance training with this dietary change has recently been shown to result in a significant hypertrophy of type IIA muscle fibres—suggesting its potential to reverse age-related effects [96]. Much more needs to be known about the effects of changing dietary patterns when combined with exercise training or increased physical activity, and the potential of this approach to protect and promote muscle health among older adults.

## 5. Evidence Summary

The aim of this review was to provide an overview of current evidence that links differences in diet and nutritional status to muscle strength, a key characteristic of sarcopenia [14], considering both variation in habitual diets in observational studies, as well as supplementation effects in intervention studies and randomised trials. A number of messages come from the reviewed evidence. Firstly, whilst many observational studies provide suggestive evidence of both higher nutrient intakes and habitual diets of higher quality being linked with greater muscle strength in older adults, findings are often inconsistent, with the benefits observed in some studies balanced by lack of effects seen in others. Secondly, trial evidence of the effects of dietary change achieved through nutrient supplementation, or changes in food consumption, is often lacking (antioxidant nutrients, LCPUFAs, whole foods and dietary patterns) or, in common with the observational studies, is inconsistent in its findings (protein, vitamin D). While there are likely to be many contributing factors to this lack of consistency, arising particularly from differences in study populations, both in observational studies and trials, as well as differences in study design, overall, there is currently little evidence from these studies of muscle strength to inform dietary recommendations for older populations. For example, there is continuing debate regarding protein requirements in older age. While essential amino acids, particularly leucine, are considered key to the anabolic response, the optimal amount and balance of animal (leucine-rich) protein vs. plant protein in the diet remains to be defined [97]. Additionally, differences in the timing/pattern of protein consumption and meal feeding also need further investigation [98,99]. However, a third message from the reviewed studies is that improvements in strength have been found in trials that combine dietary change with exercise training. Although evidence of benefit from this type of trial has often been mixed [88], and the balance of existing studies is largely from interventions based on protein/amino acid supplements, there appears to be some consistency in recent meta-analysis of this evidence. Intriguingly there is also suggestion of comparable effects in some recent studies of different types of dietary intervention (*n*-3 LCPUFAs, dietary pattern), although further research is needed. Finally, this review focused on muscle strength, and the messages may differ had other muscle outcomes been considered. For example, the meta-analysis reported by Hou and colleagues found differences in muscle strength, but not in physical performance, resulting from combined protein supplementation and exercise training [90]; conversely, the systematic review of observational studies by Bloom and colleagues suggested benefits of diets of higher quality for physical performance, but found inconsistent evidence of effects on strength [66]. In part this may be explained by the balance of evidence currently available for review, but it does highlight the need for a clear focus on individual muscle outcomes. Although physical performance is strongly linked to muscle strength [100], it is multidimensional, also involving central and peripheral nervous system function [14]. Any differences in effects according to muscle outcome may therefore be informative, indicating the need for clear delineation of dietary effects on separate aspects of muscle health.

## 6. Conclusions

There is currently insufficient evidence to be able to assess the potential of optimising diets as a way of protecting or promoting muscle strength among community-dwelling older adults. Some supplementation studies have been effective, suggesting that increases in nutrient intake may be needed by at-risk individuals. However, outside the context of malnutrition, the role of poor habitual diets is less clear. Even for protein supplementation, for which there is a significant body of research showing positive effects on muscle strength [72], there is less evidence that low habitual protein intakes are causally linked to sarcopenia in older populations [13]. For example, in a two-year supplementation study of community-dwelling older Australian women, provision of a daily high-protein drink did not change muscle mass or strength [101]. This has obvious implications for the possible translation of existing evidence to define dietary recommendations in older populations.

This review has highlighted a number of gaps in the evidence for future research to address.

A clearer understanding is needed of individual differences in responsiveness to dietary change. For example, in their systematic review and meta-analysis, Veronese and colleagues found differential effects of dietary interventions according to health status, such that protein supplementation was more effective among frail participants [72]. Also, a number of studies point to the potential of gender effects that need further exploration. Addressing this gap in understanding may help to explain the heterogeneity seen across studies and other inconsistencies in the evidence, but more importantly, could identify the need and importance of targeted nutritional support for some individuals.

For many dietary constituents, more complete information is needed to enable understanding of their effects on muscle strength. In particular, dietary messages for younger and older adults may differ; more needs to be known about the distinction between protective actions, perhaps central to future preventive strategies for younger adults, compared with therapeutic actions, which could be used for treatment of older adults who have sarcopenia. However, progress in developing this evidence may rely on future improvements in dietary assessment methodology. Traditional methods are commonly based on self-reported intakes that are burdensome, which may limit participation [102], and associated with measurement error. Novel approaches are therefore needed, including exploration of the potential of combining reported intakes with dietary biomarkers [103].

Further evidence is also needed about the role of dietary patterns and whole foods, as influences on muscle strength, to be able to evaluate the potential benefits of changes in patterns of food consumption that increase intakes of groups of nutrients and non-nutrients at the same time. For example, changing dietary patterns to a ‘healthier’ profile of foods could increase intakes of plant bioactive components as well as nutrients—an approach that would be expected to be more effective than simple supplementation with single nutrients. Such changes in diet could also have positive implications for the composition of the gut microbiota, recently linked to beneficial effects on muscle health [32].

Aligned with improving understanding of the effects of changes in dietary patterns, their interaction with exercise training needs further study. Although the limited evidence available suggests that dietary change to a healthier profile of foods, in combination with exercise training, is effective [95], other studies are needed to confirm and develop these findings. Such an approach—promoting diet quality alongside increased physical activity—would align with existing public health guidance and could be easily applied.

At present, our understanding of the role and importance of nutrition in aetiology of low muscle strength and sarcopenia is incomplete, preventing evaluation of the potential of using dietary change for prevention or treatment. However, the sound mechanistic links between nutrition and muscle function, together with some of the current evidence, suggest that further research is needed to develop understanding and enable consideration of implications for future dietary recommendations to protect and promote muscle health in older populations.
